# Fast phonetic learning in very young infants: what it shows, and what it doesn't show

**DOI:** 10.3389/fpsyg.2014.00511

**Published:** 2014-06-02

**Authors:** Ocke-Schwen Bohn, Linda Polka

**Affiliations:** ^1^English Degree Program and Center on Autobiographical Memory Research, Department of Psychology, Aarhus UniversityDenmark; ^2^School of Communication Sciences & Disorders, Centre for Research on Brain, Language and Music, McGill UniversityMontreal, QC, Canada

**Keywords:** infant vowel perception, fast phonetic learning, Natural Referent Vowel framework, perceptual asymmetry, infant MMR (mismatch response)

One of the very solid findings from infant speech perception research is that infants start out as universal perceivers and that their perception becomes attuned to the ambient language(s) mostly during the second half of the first year of life. This language-specific alignment of perceptual abilities happens early for tones (4–6 months, Yeung et al., 2013) and later for consonants (8–12 months, Werker and Tees, 1984, but see Best et al., 1988). The results for vowels are less clear-cut; some studies report language-specific discrimination by 6 months (Kuhl et al., 1992; Polka and Werker, 1994) whereas others find this pattern emerging as late as 12 months (Polka and Bohn, 1996).

The study by Wanrooij et al. (2014) is a welcome addition to the literature as it explores whether phonetic learning can occur at a very early age, and, if so, what its mechanism(s) might be. Wanrooij et al. (WBZ) examined the neural response of two groups of Dutch-learning 2-to 3-month-olds to non-native English vowels [ε] and [æ] after short exposure (12 min) to either a bimodal or a unimodal distribution of isolated steady-state vowels along an [ε-æ] continuum. Mismatch responses from these infants, whose native language has [ε] but not [æ], indicated discrimination of the [ε-æ] contrast for the bimodally-exposed but not for the unimodally-exposed infants.

WBZ conclude that short-term distributional learning impacts how young infants perceive speech sounds. This claim is well supported, interesting, and informative. A very short laboratory exposure clearly altered the infants' immediate response to speech stimuli (in some conditions). WBZ also claim that this learning mechanism generalizes to shape vowel perception outside the laboratory and can “affect vowel perception already in the first months of life.” However, several critical limitations of this study preclude this appealing but overly broad interpretation.

First, the training conditions implemented by WBZ lack the complex acoustic variability found in a natural language context. Second, their experimental manipulations cannot be directly equated with differences in language experience. WBZ describe the bimodal distribution encountered by one infant group during the 12-min training as a “native contrast,” and the unimodal distribution encountered by the other infant group as a “non-native contrast.” This is a redefinition of the terms “native” and “non-native” which is inconsistent with the literature on speech perception and which has no ecological validity. Both infant groups in the WBZ study are exposed to Dutch in which [ε-æ] is a non-native contrast; their language experience cannot be re-defined on the basis of a 12-min exposure to a set of isolated vowel stimuli from a restricted part of the vowel space. Third, it is unclear whether both training conditions simulate vowel phonetic properties in a realistic way. The study compares the effects of exposure to stimulus distributions with either two well-defined modes or a single poorly-defined mode. Specifically, the variability around the peak in the “unimodal” condition (indexed by standard deviation of formant values) is twice that of the bimodal peaks. Thus, exposure in the “unimodal” group may be more properly described as an “amodal” or flat distribution, unlike a natural vowel category. Importantly, the construction of “bimodal” and “unimodal” exposures implicitly assumes that, in this task, infants perceptually resolve all the points along the manipulated dimension; this is unlikely to be the case and data addressing the perceptual resolution of the continuum are not available. Fourth, as the authors point out, the study lacks an untrained control group; without an “unexposed” baseline the precise impact of the exposure manipulations is unknown.

WBZ also analyze their results to test predictions generated by the Natural Referent Vowel (NRV) framework as presented in Polka and Bohn (2011). According to NRV, young infants display perceptual biases favoring peripheral vowels due to formant convergence or focalization (cf. Schwartz et al., 2005). Studies employing a variety of behavioral and neurophysiological paradigms support this hypothesis (reviewed in Polka and Bohn, 2003, 2011; see also Pons et al., 2012; Dufour et al., 2013). The NRV framework makes general predictions about how perceptual biases will become shaped via long-term natural language experience. Importantly, these predictions are not about the immediate effects of controlled short-term laboratory training manipulations of the sort implemented by WBZ. Contrary to what WBZ claim, the NRV framework currently does not yield differential predictions for 2- to 3-month-olds following a 12-min exposure to artificial stimulus distributions. Specifically, NRV does not predict an asymmetrical response for the “unimodal” but not for the “bimodal” condition. Rather, NRV predicts that infants this young would show an asymmetry in discrimination of [ε]-[æ], regardless of their native language experience. The findings in the bimodal condition support this prediction, providing the first evidence of a vowel perception asymmetry in 2- to 3-month-olds. Among the four subgroups tested (bimodal [ε], bimodal [æ], unimodal [ε], unimodal [æ]), only the bimodal [ε] group had an MMR amplitude that is significantly different from zero. Thus, infants showed a reliable MMR in the bimodal condition and, consistent with NRV, only when the deviant vowel is the more peripheral and more focal [æ]. MMR amplitude differences were also noted across the standards in the “unimodal” group. However, in the unimodal group the MMR amplitudes themselves were not significantly different from zero when either [ε] or [æ] was the standard, thus, a reliable MMR to the test tokens was absent following the “unimodal” exposure, which confirms the main effect of the exposure. We conclude that WBZ's claim that their findings fail to support NRV predictions is not valid. As WBZ point out, the asymmetrical response (in the bimodal group) may or may not have been in place before the exposure conditions.

In summary, WBZ show that the neural response to speech can be altered in very young infants in the laboratory, allocating a potential role for distributional learning mechanisms in the first few months of life. *How* and *when* this mechanism operates to shape phonetic perception in natural language contexts remains a mystery. The findings of WBZ leave no doubt that this is a mystery that is well worth solving.

## Conflict of interest statement

The authors declare that the research was conducted in the absence of any commercial or financial relationships that could be construed as a potential conflict of interest.
